# Clinical and diagnostic characteristics of complex III mitopathy due to novel *BCS1L* gene mutation in a Saudi patient

**DOI:** 10.1186/s12920-022-01210-2

**Published:** 2022-03-19

**Authors:** Mansour Al Qurashi, Ahmed Mustafa, Syed Sameer Aga, Abrar Ahmad, Abdellatif El-Farra, Aiman Shawli, Mohammed Al Hindi, Mohammed Hasosah

**Affiliations:** 1 Department of Pediatrics, Neonatology Division, King Abdullah International Medical Research Centre (KAIMRC), King Saud Bin Abdul Aziz University for Health Sciences (KSAU-HS), Ministry of National Guard Health Affairs (NGHA), King Abdulaziz Medical City, Jeddah, Kingdom of Saudi Arabia; 2 Department of Basic Medical Sciences, College of Medicine, King Saud Bin Abdul Aziz University for Health Sciences (KSAU-HS), King Abdullah International Medical Research Centre (KAIMRC), Ministry of National Guard Health Affairs (NGHA), King Abdulaziz Medical City, Jeddah, Kingdom of Saudi Arabia

**Keywords:** *BCS1L* gene, Complex III, Mitopathy, Metabolic acidosis, Björnstad syndrome, Leigh syndrome, Mitochondrial disorders, Gracile disease, Case report

## Abstract

**Background:**

Of the many types of mitochondrial diseases, mutations affecting *BCS1L* gene are regarded as chief cause of the defective mitochondrial complex-III, affecting normal mitochondrial functioning, and leading to wide variety of phenotypes.

**Case presentation:**

In this case report we describe a novel genotype linked to a unique phenotype in a Saudi patient born of a consanguineous marriage. Detailed genetic analysis and whole genome sequencing identified a novel homozygous missense mutation in exon 5 c.712A > G (p.Ser328Gly) of the *BCS1L* gene, with predicted deleterious effects on the functioning AAA^+^-ATPase domain of the protein characterized by distinct clinical presentation associated with profound multisystem involvement, conductive hearing loss, absent external auditory canal, low posterior hair line, short neck, micro and retrognathia, over riding fingers, rocker bottom foot, small phallus with bilateral absent testis (empty scrotum) and intolerable lactic acidosis.

**Conclusions:**

A pathogenic effect of this novel *BCS1L* mutation was reflected in the patient with his failure to thrive and a complex clinical and metabolic phenotype.

## Background

Among numerous genetic disorders which are known to affect humans, mitochondrial genetic defects occupy a unique position in not only being rare but also in having a wide variety of phenotypic affects [1]. Mutations in either mitochondrial (mtDNA) or nuclear genome which lead to the deficiency of mitochondrial electron transport chain (mETC) has been implicated in the pathogenesis of a wide range of neurological disorders affecting both adults and children [1–3].

Complex III of mETC (CIII, EC 1.10.2.2), also termed as cytochrome bc1 complex, plays a central role in metabolism as it is responsible for oxidizing coenzyme Q and reducing cytochrome c while doing dual functions of transporting electrons as well as pumping proton out of matrix into the intermembrane space [3]. Located within the inner mitochondrial membrane, CIII is made of a total 11 subunits—10 of which are coded by nuclear genes and one by mitochondrial DNA [3, 4]. The most frequent deficiency of mitochondrial respiratory chain occurs due to the mutations affecting the Bcs1 homolog, also known as ubiquinol-cytochrome c reductase complex chaperone (BCS1L) gene [1, 5–8].

*BCS1L* gene, located on chromosome2q35, is a member of the AAA^+^ (ATPases associated with various cellular activities) family. This gene encodes a 419-amino-acid mitochondrial chaperone protein (BCS1L) which is required for the assembly of mitochondrial CIII’s Rieske iron-sulfur subunit [1, 9]. Till date, over 100 different variants of *BCS1L* gene have been reported on clinVar (https://www.ncbi.nlm.nih.gov/clinvar/) while as the Human Gene mutation database categorizes 48 unique mutations for *BCS1L* gene [10, 11] (HGMD, 2022; NCBI, 2022). Out of these variants 42 have been categorized as likely pathogenic mutations [1, 8, 12, 13]. All of which have been reported to disrupt the overall structure of CIII, thereby resulting in the reduction of the enzymatic activity of the respirasome and concomitantly increasing the production of reactive oxygen species (ROS’s). As cellular mechanism adapts to acclimatize to the decreased function of CIII with increasing mitochondria genesis, the production and effects of ROS’s become more pronounced in the affected individual [14].

The clinical manifestations of *BCS1L* gene mutations vary widely in respect to different tissue involvement and disease progression [5, 6, 15, 16]. Generally, these mutations are associated with three kinds of disease phenotypes which are as: (a) Björnstad syndrome (OMIM: 262,000) characterized by highly restricted and abnormal flat twisted hair shafts (*pili torti)* and sensorineural hearing loss (b) a profound multisystem organ failure identified by severe mitochondrial complex III deficiency (Online Mendelian Inheritance in Man, OMIM: 124,000) presenting with encephalopathy of variable severity, tubulopathy, encepthalopathy and/or hepatomegaly and (c) a multivesicular syndrome characterized by sentinel growth retardation, aminoaciduria, cholestasis, iron overload, lactic acidosis, and early death referred to as GRACILE syndrome (OMIM: 603358) [5, 14, 17–19]. Additionally, BCS1L mutations has also been implicated in the Leigh Syndrome (OMIM: 256000) characterized by psychomotor and mental regression followed by death within few years of birth [9, 17, 20, 21].

GRACILE syndrome was historically defined back in 1998, as a metabolic disorder with an autosomal recessive mode of inheritance in which infants did not have any *pili torti* or deafness [22]. So far it has been reported mostly in newborn infants with parents of Finnish, British, Spanish, Caucasians, Turkish, Kenyan and Saudi origin [9, 18, 19, 23–28]. Furthermore, it has been reported to result in the isolated fatal mitochondrial encephalopathy [15] and a syndrome of neonatal tubulopathy, encephalopathy, and liver failure [9]. Most reported cases of lactic acidosis due to *BCS1L* mutations usually have an early-onset and is known to be fatal causing early death [8, 21, 29].

Here, we report a novel homozygous mutation in the *BCS1L* gene in a patient from a distinct consanguineous Saudi family. The patient displayed a severe neonatal metabolic acidosis and persistent high creatine kinase with hyper uricemia with elevated liver enzymes.

## Case presentation

### Clinical investigations

Soon after birth, patient was subjected to a new born screening as per the protocol followed by the NGHA, which included evaluation of structural and metabolic anomalies. Liver, kidney and metabolic panels were ordered to be assess the organ and overall health. Assessment of aminoacidemia, aminoaciduria, lactic acidosis, and metabolic acidosis was also performed.

### Whole exome sequencing

After proper sessions of genetic counselling and explanation of the conditions, parents of the patients consented to all testing procedures including Genome Wide Study (GWS) of their and patient’s genomes. Commercially available whole exome sequencing (WES) and analysis were performed on the blood samples by Bioscientia International (Ingelheim, Germany). Genomic DNA from the patient was fragmented and the coding exons of the more than 20000 genes of patient’s DNA were enriched using Roche KAPA capture technology (KAPA HyperExome Library), amplified and sequenced simultaneously by Illumina technology (next-generation sequencing, NGS) using illumine system, for studying the recessive, X-linked and dominantly inherited diseases. The target regions were sequenced with an average coverage of 149-fold.

### In silico analysis and pathogenicity

Although, the commercially available NGS reported that 18 out of 22 bioinformatics in silico programs predicted a pathogenic effect for this variant. We still did an independent analysis for the prediction of the impact of this mutation was also done using commonly available bioinformatics tools PROVEAN (Protein Variation Effect Analyzer: http://provean.jcvi.org/index.php), PolyPhen-2 (Polymorphism Phenotyping v2: http://genetics.bwh.harvard.edu/pph2/), and SIFT (Sorting Intolerant From Tolerant: https://sift.bii.a-star.edu.sg/www/SIFT_seq_submit2.html). Three-dimensional structure of pG238G mutation at ATP binding site of the BSC1L protein was predicted by SWISS-MODEL homology modeling.

## Results

### Clinical report

Here we report a male infant having a novel homozygous *BCS1L* gene mutation and having a distinct clinical presentation associated with profound multisystem involvement and intolerable lactic acidosis.

**Fig. 1:**
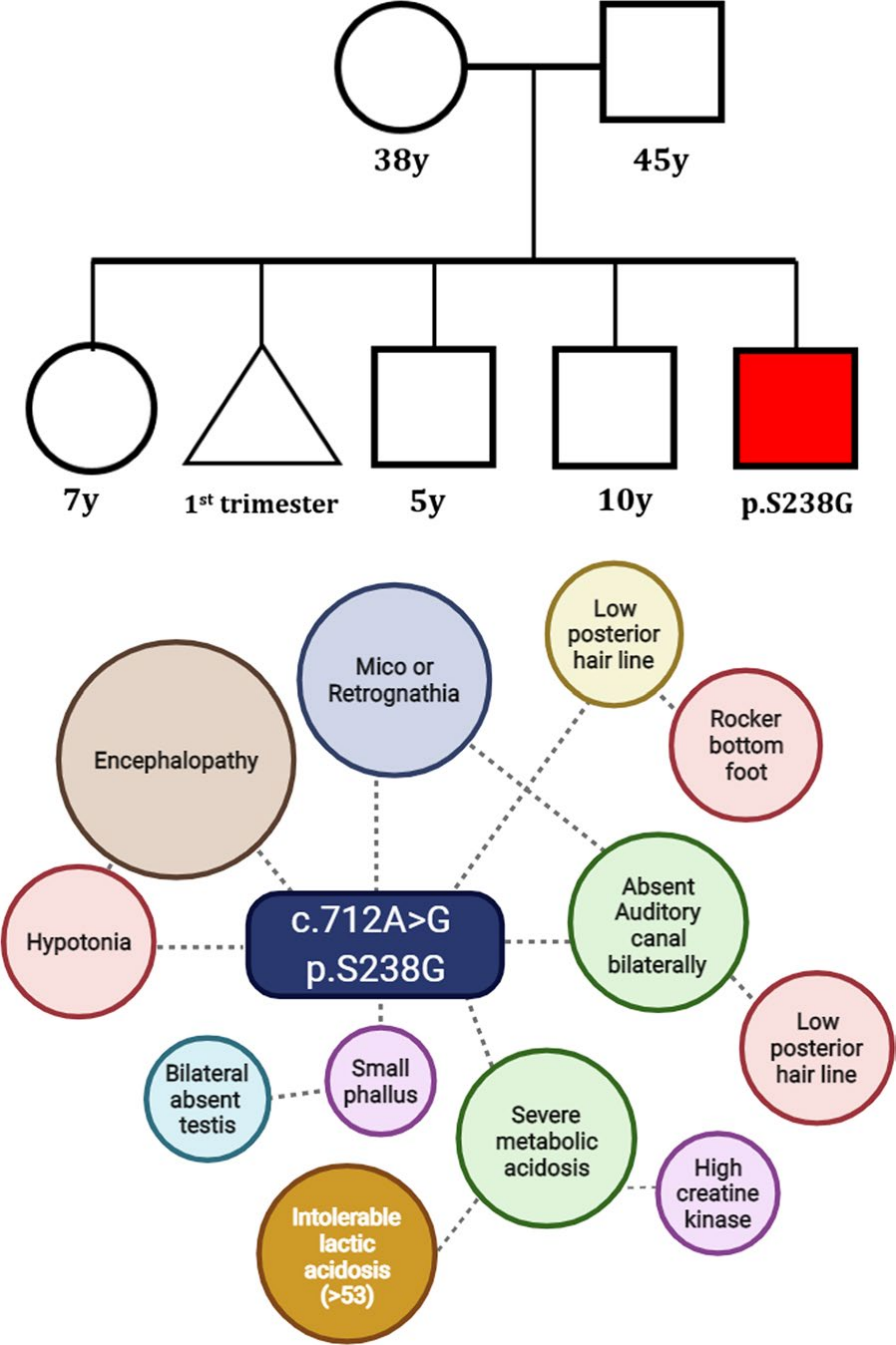
Pedigree and the clinical characteristics of the patient. The parent had one spontaneous miscarriage of second pregnancy in 1st trimester and earlier born three children (2 M and 1F) born are normal

This infant was the fifth offspring of consanguineous Saudi parents (Fig. 1), who was born at a late preterm gestation (36 weeks) via emergency caesarian section, following a high resistant umbilical artery Doppler suggesting growth restriction at 34 weeks (weight = 1.965kgs). The pregnancy was smooth apart of intrauterine growth restriction and the birth weight was 1840 g (< 10th centile), length 41.5 cm (< 10th centile) and head circumference 34.5 cm (> 50th centile). APGAR Score was 3@1 min, 5 @5 min, 5@ 10 min which did not improve further at 15 and 20 min.

Mother was G5P3 + 1 and of 38 years old at the time of this pregnancy. She had a history of total thyroidectomy eight years ago due to benign Hurthel cell adenoma, and was on treatment with L thyroxine as hypothyroidism and 1 alpha with calcium supplement as hypoparathyroidism. She has 3 living healthy children all delivered through caesarian sections. One miscarriage was spontaneous in the first trimester of the second pregnancy.

Baby delivered flat, with an initial heart rate < 100 bpm and after initial steps of resuscitation on Positive Pressure Ventilator (PPV) through face mask, the heart rate had improved subsequently above 100 bpm but the baby was limpic and hypotonic with no obvious spontaneous breaths or movements. Intubation at 7 min with size 3 endotracheal tube. The HR further improved to 120/ min with SPO_2_ 92%.

**Fig. 2:**
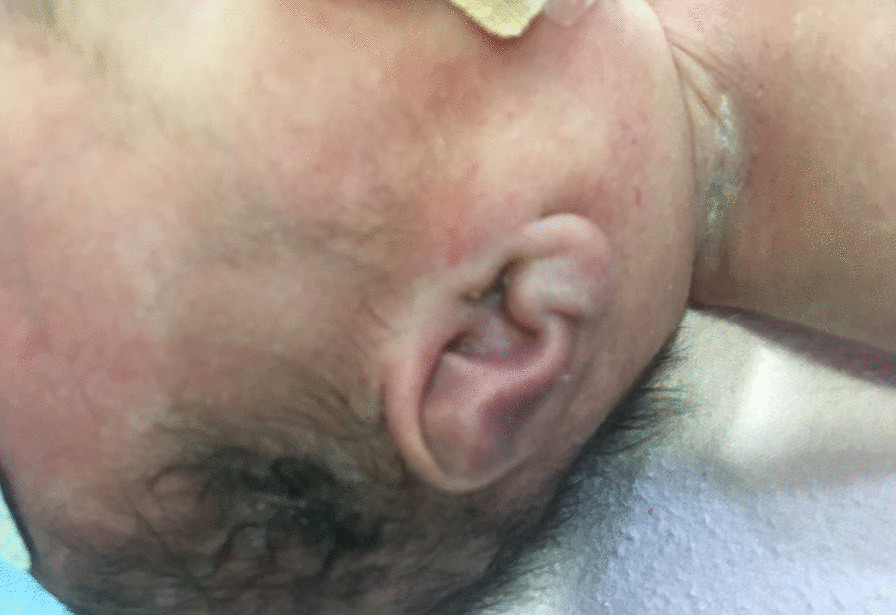
The prominence of dysmorphic features in form of low set posteriorly placed ears

**Fig. 3:**
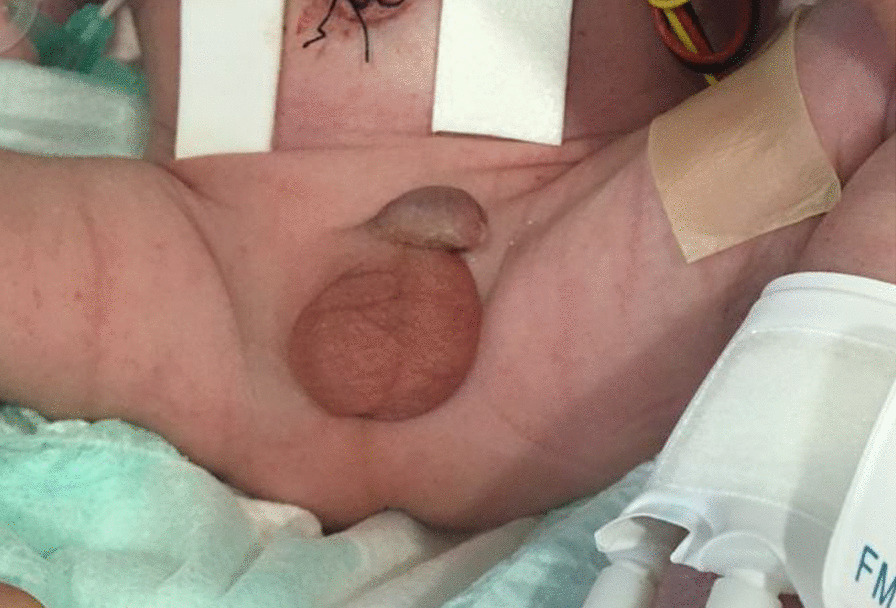
The small phallus with bilateral absent testis (empty scrotum)

**Fig. 4:**
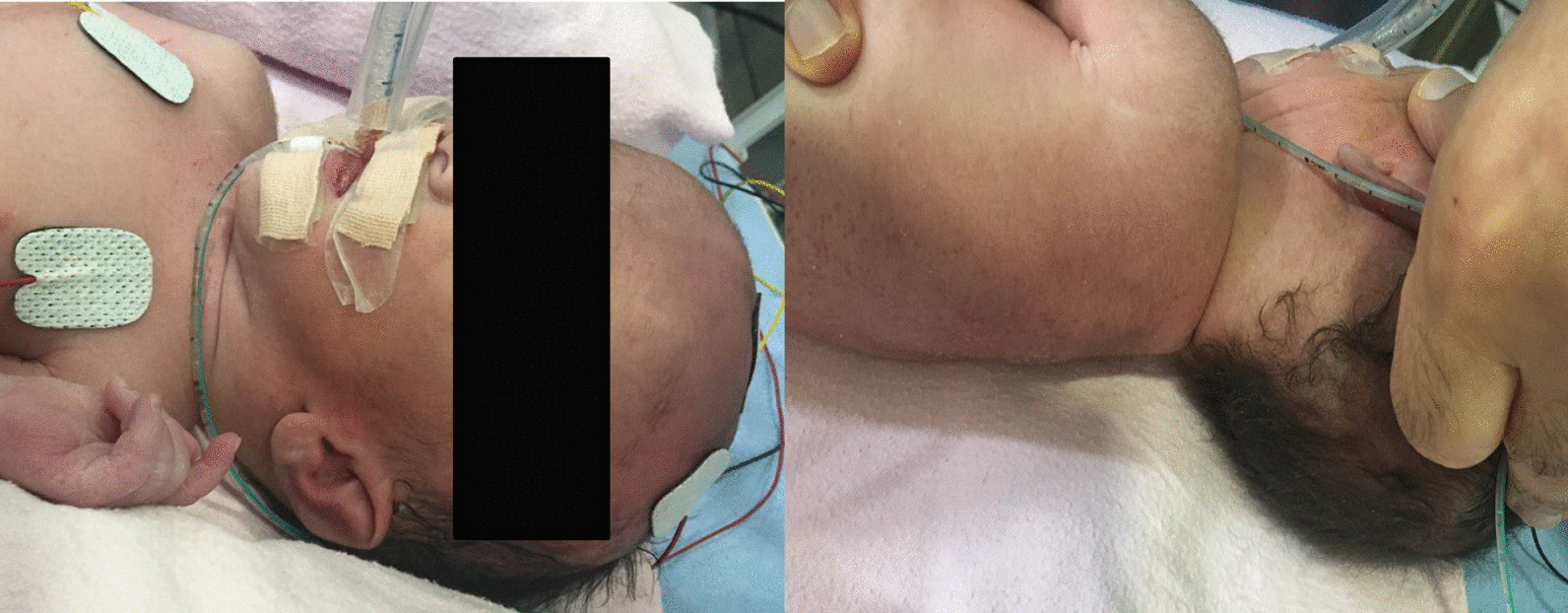
Showing dysmorphic features in form of high arched palate, low posterior hair line, short neck, micro and retrognathia, over riding fingers

Initial examination of the infant revealed that he had significant dysmorphic features in form of low set posteriorly placed ears, absent external auditory canal bilaterally, hypertelorism, high arched palate, low posterior hair line, short neck, micro and retrognathia, over riding fingers, rocker bottom foot, small phallus with bilateral absent testis (empty scrotum) (Figs. 2, 3, 4). The features were look alike infants with Trisomy 18 (Edwards Syndrome).

Central nervous system examination revealed that the baby was in deep coma, with profound encephalopathy and no response to painful stimuli, bilateral reacting pupils, severe hypotonia. Cardiovascular examination shows borderline tissue perfusions with palpable femoral pulsation and no audible murmur. Respiratory system was supported by mechanical ventilator as the infant had inefficient respiratory drive and abdominal examination was unremarkable.

He had a severe metabolic acidosis since birth (PH ranging 7.11–6.99; bicarb (HCO_3_−) 12 to 8.9 and BE − 20 to − 16). He had high concentration of creatine Kinase (= 340) with hyper uricemia (= 443) and refractory metabolic acidosis, and elevated liver enzymes. The amino acid profile was also high throughout his four days before death (Tables 1, 2, 3). The Lactic acid levels were persistently high (> 53) until he died.

**Table 1:** Blood biochemistry of the patient

	Values at day 1	Values at day 2	Reference range
BUN	2.6	5.9	1.0–8.2 mmol/L
Sodium	147	183	135–144 mmol/L
Potassium	3.7	3.9	2.5–4.9 mmol/L
CO2	8	9	5–20 mmol/L
Chloride	97	87	101–111 mmol/L
Bili T	75.8	66.7	3.3–11.7 umol/L
Alk Phos	176	122	90–273 U/L
Creatinine Level	38	79	37–93 mmol/L
Phos	1.77	2.83	1.80–3.40 mmol/L
Ca	1.95	1.62	2.13–2.74 mmol/L
Cholesterol Total	1.10	0.26	1.2–3.23 (F)/1.1–2.82 (M) mmol/L
GGT	287	140	23–219 mmol/L
Glucose Random	3.1	3.8	2.8–4.4 mmol/L
Trig	1.21	3.72	0.93–2.93 mmol/L
TP	34	21	53–83 g/L
Mg	0.79	0.90	0.82–1.62 mmol/L
Albumin Level	20	13	33–45 g/L
Lactic Acid	14.68	53	0.7–2.0 mmol/L
Ammonia	68	89	56–107 umol/L
Uric Acid		443	164–757 umol/L
Creatine Kinase		3404	27–132 (F)/45–200 (M) U/L

*BUN* Blood Urea Nitrogen, *CO*_2_ Carbon Dioxide, *Bili T* Total Bilirubin, *Alk Phos* Alkaline Phosphate, *Phos* Phosphate, *Ca* Calcium, *Trig* Triglycerides, *TP* Total Protein, *Mg* Magnesium

**Table 2:** Blood gases of the patient

	1 h	12 h	Day 2	Day 3	Reference range
pH	7.059	6.99	6.758	6.973	7.35–7.45 (F)/ 7.34–7.45 (M)
pCO2	44.7	52.0	96.2	49.8	32–45 (F)/35–48 (M) mmHg
pO2	127.4	63.5	50.6	89.8	83–108 mmHg
HCO3	12.3	12.3	13.3	11.3	20–24 (F)/22–26 (M) mmol/L
BE	17.8	− 19.4	− 21.9	− 20.0	− 3.3 to 1.2 (F)/ − 2.4 to 2.3 (M) mmol/L

**Table 3:** Serum amino acid levels at day 2 of birth

Amino acid	Levels in umol/L	Reference range (unit)
Taurine	154	14–238
Aspartic acid	15	1–21
Threonine	204	53–141
Serine	137	62–206
Asparagine	109	38–114
Glutamic acid	89	32–104
Glutamine	775	198–886
Glycine	1292	101–317
Alanine	1373	108–448
Citrulline	< 5	5–33
Valine	237	65–201
Cystine	17	20–60
Methionine	86	6–50
Isolucine	85	22–82
Leucine	165	47–175
Tyrosine	317	38–178
Phenyalanine	103	21–85
Orthinine	65	31–207
Lysine	517	67–291
Histidine	117	25–113
Arginine	31	12–116
Proline	940	120–344

### Whole exome sequencing

**Fig. 5:**
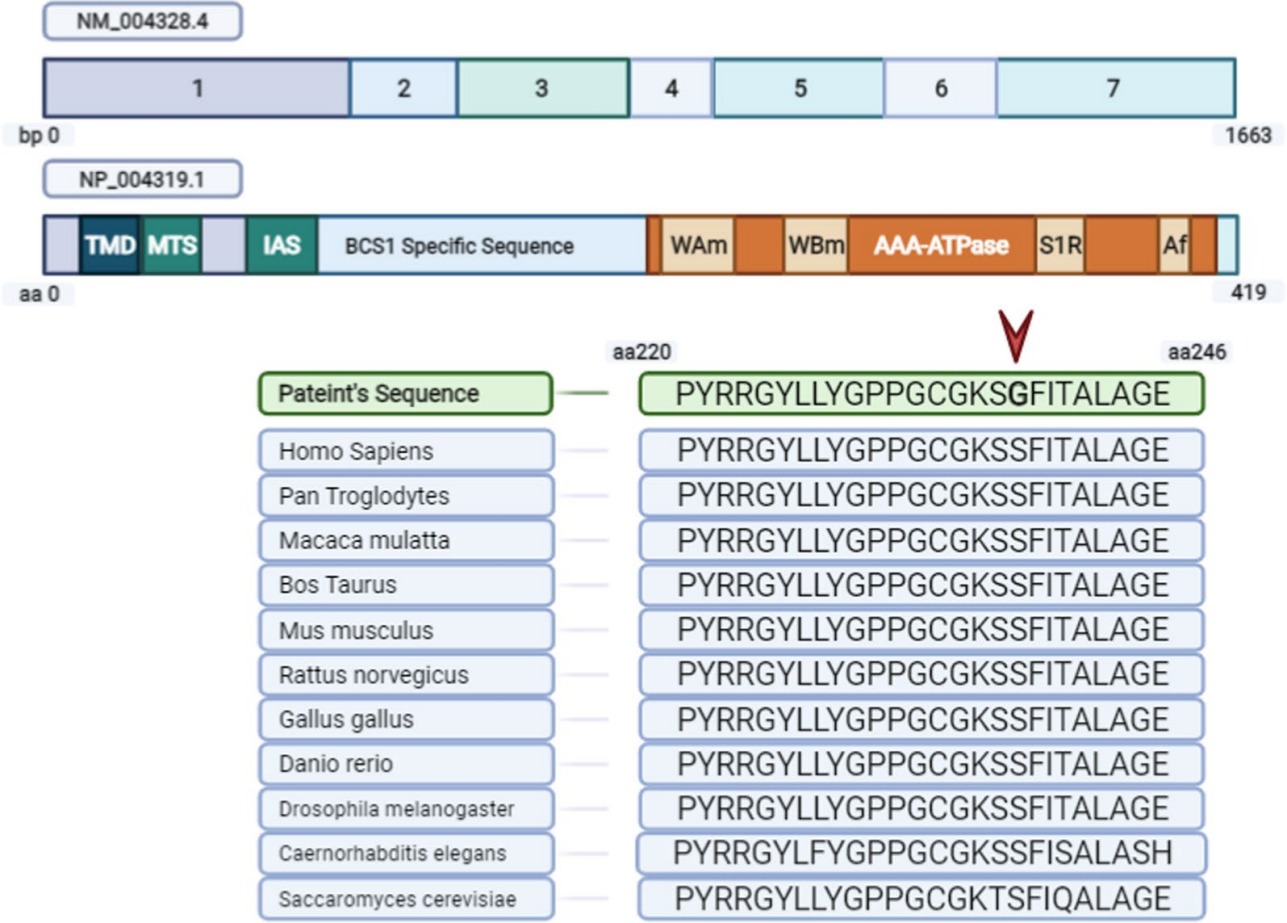
Schematic representation of BCS1L gene (seven exons) and protein (419 amino acids), denoting the positions of essential domains. The position of the BCS1L mutation reported in the present study is indicated with an arrow (S238G). For comparisons, partial amino acid sequence alignments of BCS1L in different species is shown highlighting the evolutionary conservation

**Fig. 6:**
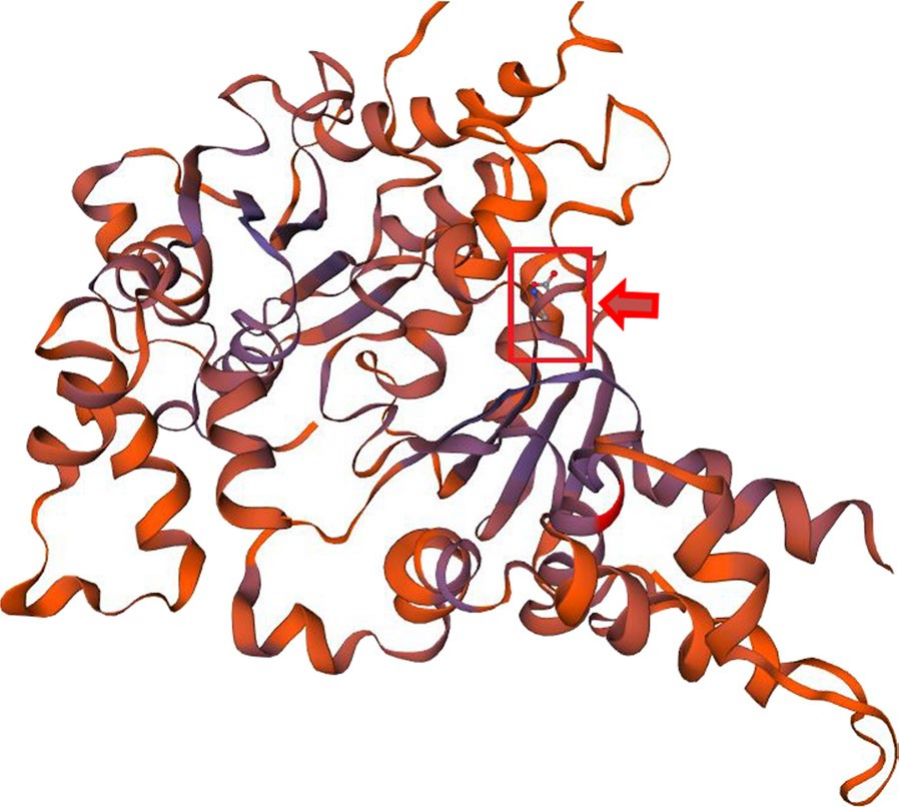
The three dimensional SWISS-MODEL structure of a monomer of BCS1L protein. The predicted SWISS-MODEL secondary structure of BCS1L protein with Glycine 238 residue being highlighted within red box. The alpha helices are shown as red-cylinders, beta sheets as purple arrows and random coils as lines/ribbons

**Fig. 7:**
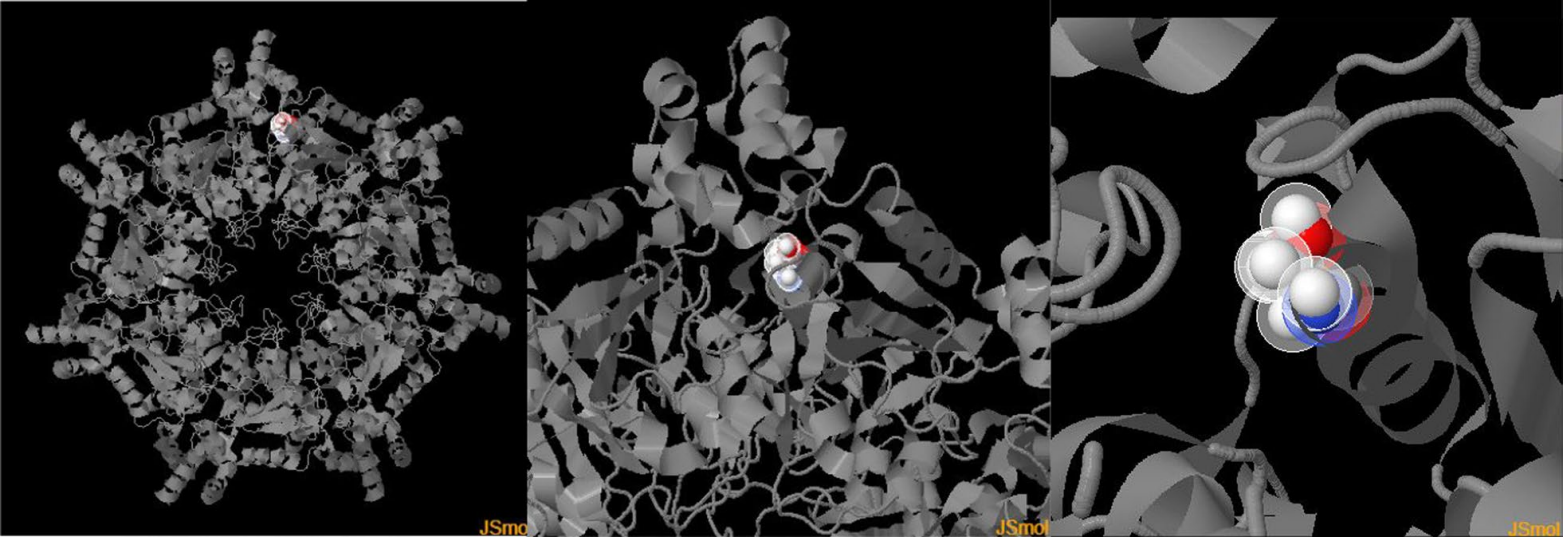
The three dimensional PolyPhen-2 structure of a hexamer of BCS1L protein. The 238Glycine residue being highlighted in white/red balls at the top mid area

WES identified a homozygous variant c.712A > G (p.S238G) in *BCS1L* gene (OMIM: 603647). The mutation was spontaneous and a novel, not reported in literature so far. The mutation affected the exon 5 of the *BCS1L* gene, which is known to code for the highly conserved domain of the protein called as AAA^+^-ATPase (Figs. 5, 6, 7).

### In silico analysis and pathogenicity

All three bioinformatics tools i.e., PROVEAN, PolyPhen-2, and SIFT predicted the pathogenic and deleterious nature of the mutation. SWISS-MODEL of the monomeric BCS1L protein showing the mutation site is shown in Fig. 7.

## Discussion and conclusions

Among the genetic disorders mitochondrial diseases occupy a special place which are characterized by the varied defects in the oxidative phosphorylation function of the organelle [29]. These mitochondrial disorders (MDs) are caused by pathogenic mutations either in the nuclear DNA (nDNA) or in mitochondrial DNA (mtDNA) that encode structural mitochondrial proteins or proteins involved in mitochondrial function [1, 29]. They make up the most common group of inherited metabolic disorders and the most common forms of inherited neurological disorders [4, 6, 29].

Most of the MDs directly or indirectly affect the metabolism because of the deficiency of critical oxidative phosphorylation (OXPHOS) components. Mitochondrial electron transport chain (mETC) consists of five multi-subunit complexes, of which complex III deficiency is caused by mutation in *BCS1L* gene (OMIM: 603647), is relatively rare among all reported MDs [30]. BCS1L protein coded by *BCS1l gene* serves as a chaperone/translocase in the inner mitochondrial membrane, where it functions to facilitate the final folding and assembly of the complex III by properly inserting the Rieske Fe/S protein into it [1, 16].

BCS1L in its functional state consists of three distinct domains: (a) N-terminus containing three specialized parts, i.e., transmembrane domain (TMD), mitochondrial targeting sequence (MTS) and import auxiliary sequence (IAS), (b) a BSC1L-specific domain and (c) an AAA-ATPase domain at the C-terminus (Fig. 5). The TMD/MTS interaction with BCS1L helps in the anchoring the protein and its consequent transport within the mitochondrial matrix [21, 31]. ATPase domain of BCS1L spans from amino acids 220–419 and is actually classified as P-loop NTPase, and usually such ATPase exists in functional state as hexamers. Additionally, within the ATPase domain itself, there are four highly conserved structural features, which include Walker A motif (WAm), Walker B motif (WBm), Sensor 1 Region (S1R) and an Arginine finger (Af) [32] (See Fig. 5). In Current case, the patient has a missense mutation in exon 5 at c.712A > G (transition) corresponding to 238 codon of the gene resulting in ser to glycine amino acid change (p.S238G). This is the novel mutation not reported so far in literature. This mutation is present within a highly conserved AAA-Family ATPases domain of the BCS1L protein, and as predicted by insilico analysis, the mutation being deleterious results in the alteration of the normal functioning of the protein, whose interaction with the other proteins remains constitutively defective (See Figs. 5, 6, 7).

Numerous similar mutations affecting the AAA-ATPase domain of the BSC1L protein have been documented in literature which include—c.838C > T (p.L280F) [21]; c.703G > A (p.G235R) [33]; c. 688G > C (p.G230R), c755G > A (p.C252Y), c.785_786del CT (p.Ser262*), c.919C > T (p.L307F), c.1220_1220delC (p.P407L-fs*2) & c.1250 T > C (p.L417P) [8]; c.830G > A (p.S277N) [9]; (p.R291Ter), (p.Q302E), & (p.R306H) [14]; and c.980 T > C (p.Val327A), c.1057G > A (p.V353M), & c.1102 T > A (p.F368I) [15, 17]. All of these mutations have been demonstrated to fall within or are adjacent to each other in the three dimensional structure of the protein and most likely alter the activity of BCS1L; leading to a phenotype that fall in the BCS1L mitopathy category [8, 21, 32].

Additionally, most of these mutations have been found to have a genetic etiology of mosaicism of two diseases representing both Björnstad and GRACILE syndrome with sentinel signs of growth retardation, aminoaciduria, cholestasis, iron overload, lactic acidosis, and early death as well as sensorineural hearing loss, pili torti, nodous trichorrexis [8, 33]. And as documented and suggested earlier by various researchers [1, 9, 21], our case also falls in the category of an intermediate BCS1L myopathy involving a combination of clinical characteristics [8, 14, 15].

Furthermore, this novel mutation (p.S238G) falls within the Walker A motif of the AAA-ATPase domain as does the p.S235R and p.G230R reported previously [8, 21, 33]. Walker A motif is an essential part of AAA-ATPase domain located adjacent Walker B motif in the three dimensional and in necessary for ATP binding and interaction for carrying out its constitutive reactions [21, 34]. Hikmat et al. [8] previously reported that p.G230R mutation in Walker A motif of ATPase domain to be extremely destabilizing due to severe amino acid clashes hampering the proper folding of the protein to its functional state, while Falco et al. [33] reported that p.S235R mutation located in proximity of ATP binding site, result in the altering of the ATP binding affinity of the domain for its effectors i.e., ATP/ADP. Baker et al. [21] also reported that the novel p.L280F mutation found in the Walker B motif of the AAA-ATPase domain fall into the BCS1L Mitopathy category and shows similar phenotypic characteristics as previously reported for p.S277R mutation like lactic acidosis, developmental delays, hearing loss etc. [9].

Classically, GRACILE syndrome patients from Finland were reported homozygous for a c.232A > G mutation in exon 2 of the *BCS1L* gene, resulting in a substitution of serine with glycine (p.S78G) [17]. They had a normal complex III activity and no neurologic problems but did have marked iron overload. Additionally, most reported cases of lactic acidosis because of the BCS1L gene mutations are characterized by early-onset disease which is fatal and results in early death [25]. Neonatal lactic acidosis is one of the sentinel characteristics of the primary Mitopathy and has been reported in almost all cases in literature, which is considered a poor prognostic factor [1, 8, 9, 14, 15, 17, 21]. In our case there was a persistent high level of lactic acid in patient (> 53) until he expired together with the high levels of creatine kinase, uric acids and almost all amino acids (Tables 1, 2, 3). Furthermore, it has been also reported that that the clinical presentation in gracile syndrome is almost always associated with neonatal tubulopathy with hepatic insufficiency [1, 19, 28], which is reflective in our case also (GGT > 140–287; TP < 21–34). Tuppen et al., [25] has also reported that p.G129R *BSC1L* gene mutation presented with seizures, optic atrophy, and isolated CIII deficiency, but had normal intellect and blood lactate.

In Turkish patients, a compound heterozygosity was reported for 2 mutations in the *BCS1L* gene: a c.464C > G transversion in exon 3, resulting in an arg to pro (p.R155P) substitution, and a c.1057G > A transition in exon 7, resulting in a val to met (p.V353M) substitution [9]. In two Spanish siblings, a compound heterozygosity for mutation in the BCS1L gene was identified: one a c.246C > T transition in exon 1, resulting in an arg to cys (p.R45C) substitution, and a c.279C > −T transition in exon 1, resulting in an arg to ter (p.R56X) [35]. In Saudi population a variant phenotype has been reported which was associated with a missense mutation at c.385G > A; causing Gly to Arg substitution in BCS1L protein (p.G129R) [25, 28]. As reported previously by Al-Owain et al., [28] that autosomal recessive inherited (nuclear) group of mitochondrial disorders is more prevalent in Saudi Arabia because of the high rate of consanguineous marriages, the tribal family structure, and the large family size. In this case report we reported a novel homozygous missense mutation in exon 5 (c.712A > G) of the *BCS1L* gene, which resulted in the deficiency of mitochondrial complex III proteins, with distinct clinical presentation associated with profound multisystem involvement, short neck, micro and retrognathia, over riding fingers, rocker bottom foot, small phallus with bilateral absent testis (empty scrotum) and intolerable lactic acidosis.

Since the mutational spectrum of the BCS1L gene is varied which also differs among populations geographically, coupled with an obscure relationship between genotype and phenotype, the defining of the BCS1L mutation into well-defined phenotypes is proving challenging [5, 6, 8, 18, 26, 28]. Furthermore, the limited number of published cases, large number of unreported clinical features and varied clinical feature which are sometime overlapping there is a need to define the BCS1L mutation as such as mitopathies, until an exhaustive protein study will be able to connect a particular genotype with its phenotype. We concur the findings of other researchers especially few related studies [8, 20, 21, 26, 28, 33] and do vehemently propose that *BCS1L* mutation screening should be routinely included in the differential diagnosis of severe renal insufficiency in the newborn screening owning to the fact that there is a marked clinical heterogeneity associated with the mutation in *BCS1L* gene as well.

## Data Availability

The WES of the mutant BCS1L gene reported in the current study has been submitted in the ClinVar for public access (URL: https://www.ncbi.nlm.nih.gov/clinvar/variation/1341952/). Some of the unpublished and raw data has been made available for readers on FigShare (https://doi.org/10.6084/m9.figshare.19180814).

## Abbreviations

BCS1L: Bc1 synthesis like (BCS1L) gene
GRACILE: Growth retardation, aminoaciduria, cholestasis, iron overload, lactic acidosis, and early death
mtDNA: Mitochondrial DNA
OMIM: Online Mendelian Inheritance in man
MDs: Mitochondrial disorders
OXPHOS: Oxidative phosphorylation
mETC: Mitochondrial electron transport chain
TMD: Transmembrane domain
MTS: Mitochondrial targeting sequence
IAS: Import auxiliary sequence
